# Disease mechanism for retinitis pigmentosa (RP11) caused by missense mutations in the splicing factor gene *PRPF31*

**Published:** 2008-04-18

**Authors:** Susan E. Wilkie, Veronika Vaclavik, Huimin Wu, Kinga Bujakowska, Christina F. Chakarova, Shomi S. Bhattacharya, Martin J. Warren, David M. Hunt

**Affiliations:** 1 University College London Institute of Ophthalmology, London, United Kingdom; 2 Moorfields Eye Hospital, London, United Kingdom; 3 Department of Biosciences, University of Kent, Canterbury, Kent, United Kingdom

## Abstract

**Purpose:**

Missense mutations in the splicing factor gene *PRPF31* cause a dominant form of retinitis pigmentosa (RP11) with reduced penetrance. Missense mutations in *PRPF31* have previously been shown to cause reduced protein solubility, suggesting insufficiency of functional protein as the disease mechanism. Here we examine in further detail the effect of the A216P mutation on splicing function.

**Methods:**

Splicing activity was assayed using an in vivo assay in transfected mammalian cells with rhodopsin (*RHO*) and transducin (*GNAT1*) splicing templates. Pull-down assays were used to study the interaction between PRPF31 and one of its cognate partners in the spliceosome, PRPF6.

**Results:**

Splicing of *RHO* intron 3 and *GNAT1* introns 3–5 mini-gene templates was inefficient with both spliced and unspliced products clearly detected. Assays using the *RHO* minigene template revealed a direct negative effect on splicing efficiency of the mutant. However, no effect of the mutation on splicing efficiency could be detected using the longer *GNAT1* minigene template or using a full-length *RHO* transcript, splicing of which had an efficiency of 100%. No unspliced *RHO* transcripts could be detected in RNA from human retina. Pull-down assays between PRPF31 and PRPF6 proteins showed a stronger interaction for the mutant than wild type, suggesting a mechanism for the negative effect.

**Conclusions:**

Splicing of full-length *RHO* is more efficient than splicing of the minigene, and assays using a full-length template more accurately mimic splicing in photoreceptors. The RP11 missense mutations exert their pathology mainly via a mechanism based on protein insufficiency due to protein insolubility, but there is also a minor direct negative effect on function.

## Introduction

Retinitis pigmentosa (RP) represents a large, heterogeneous group of generally progressive retinal diseases that initially affect the rod photoreceptors but subsequently involve both peripheral and central cones, frequently leading to total blindness. With an incidence of 1 in 3,500 of the population, inheritance may be X-linked, autosomal dominant, or autosomal recessive. In many cases, the disease gene encodes a component of phototransduction or allied processes with expression confined to the retina, for example the rod visual pigment (reviewed in [1]), or peripherin-RDS [2], but not all RP genes show such a restricted pattern of expression. In particular, mutations in four RNA splicing factor genes underlie some autosomal dominant forms of RP, namely RP9 (*RP9*, OMIM 607331 [3]), RP11 (*PRPF31,* OMIM 600138 [4]), RP13 (*PRPF8,* OMIM 600059 [5]), and RP18 (*PRPF3*, OMIM 607301 [6]). An intriguing aspect to the association of these genes with retinal disease is that splicing occurs in every cell of the body so the genes must have a general housekeeping function, yet the disease pathology is restricted to the rod photoreceptors of the retina.

Mutations in *PRPF31* are one of the most common causes of autosomal dominant RP, with over 30 mutations reported, including missense, deletion, insertion and splice site alterations [4,7-11]. Whereas mutations in *RP9, PRPF3,* and *PRPF8* are fully penetrant, mutations in *PRPF31* are not, with several heterozygous carriers showing no deleterious visual symptoms and only slightly abnormal electroretinograms [12]. Studies conducted on *PRPF31* mRNA levels in lymphoblast cell lines isolated from family members with either deletion or splice site mutations indicate that penetrance requires the co-inheritance of a low expressing wild-type (WT) allele alongside the mutant allele [13,14], whereas the presence of a high expressing WT allele is protective. The disease phenotype would appear to arise therefore only when the level of WT splicing factor falls below a critical threshold in rod photoreceptors.

Our previous work [15,16] has investigated the functional consequences of missense mutations in *PRPF31* [4]. With missense mutations, the mutant protein is almost certainly produced so the simple answer that the disease arises solely from a haploinsufficiency of splicing factor protein may not provide a full explanation for the disease mechanism. Using a yeast complementation assay, we have previously demonstrated [15] that the introduction of the human A216P mutation into the yeast ortholog *PRP31p* results in reduced growth; the effect of the mutation would appear to be therefore to modify or reduce the activity of the protein rather than to abolish it completely. In addition, the mutant protein appears to be nontoxic in yeast even when highly expressed. Protein localization studies in mammalian cells expressing His-tagged PRPF31 showed mislocalization of the mutant proteins, with a substantial proportion remaining in the cytoplasm rather than translocated to the nucleus [15]. Further investigations revealed that this mislocalization was unlikely to arise from a deficiency in trafficking of the mutant proteins to the nucleus, but arose rather from an increased tendency for the mutant proteins to form aggregates in the cytoplasm [16]. The consequence of this is a reduction in the levels of PRPF31in the nucleus: an insufficiency of functional protein in the nucleus may be therefore the primary cause of the disease.

Our original in vivo assays of splicing function in HEK 293 cells using a bovine rod opsin splicing template failed to detect any effect of missense mutations on splicing efficiency [15]. However, Yuan et al. [17] and Mordes et al. [18] have shown a specific negative effect of two deletion mutations of *PRPF31* on the efficiency of splicing of certain introns in artificial human minigenes, in particular intron 3 of human rod opsin (*RHO*), intron 1 of *RDS* and intron 3 of *FSCN2*. Another study by Rivolta et al. [13] failed however to find any evidence for a generalized RNA splicing defect caused by various null-type mutations.

All four splicing genes linked to autosomal dominant RP encode protein factors required for the formation of stable U4/U6 snRNPs and for assembly of the U4/U6+U5 tri-snRNP. Binding of this tri-snRNP to the presplicing complex represents a key step in the formation of the spliceosome proper. PRPF31 binds to the U4 snRNP in the U4/U6 di-snRNP [19,20] and is thought to form a bridge between the U4/U6 di-snRNP and U5 by binding to the U5-specific PRPF6 (also known as U5–102 kD protein) [21]. This was confirmed by RNAi studies in which it was shown that knock-down of either PRPF31 or PRPF6 inhibited tri-snRNP formation and led to the accumulation of U5 and U4/U6 [22].

In this study, we have refined our splicing assay system to examine the effects of the A216P missense mutation in *PRPF31* on several splicing templates that include *RHO* intron 3 in a minigene construct plus two templates that more accurately reflect the real life situation, a full-length human *RHO* gene that includes introns 1–4, and a partial rod α-transducin (*GNAT1*) gene with introns 2–5. Since any direct effect of splicing factor mutations on splicing efficiency would most likely act via interactions with other components of the spliceosome, we have examined the interaction via pull-down assays of WT and mutant PRPF31 with PRPF6, the other protein component of the tri-snRNP not yet implicated in RP.

## Methods

### Plasmid constructs for splicing assays

For the rod opsin minigene assays, an exon 3 to exon 4 fragment (E3-E4) from the human *RHO* was amplified from human genomic DNA using *Nhe*I- and *Cla*I-tagged forward and reverse primers (5′-GCC GGC TAG CAT GTA CAT CCC CGA GGG CCT G-3′ and 5′-CGG CAT CGA TTC ACT TGT TCA TCA TGA TAT AGA TGA C-3′, respectively) and KOD (Thermococcus kodakaraensis) polymerase (Novagen, Nottingham, UK). This was cloned into the vector pTandem-1 (Novagen) via the appropriate restriction sites. The *PRPF31* cDNA sequence (WT or A216P) was then transferred from a corresponding pTriEx-1 construct [15] as a *Sac*II-*Xho*I fragment to generate the construct pTan.PRPF31^His^.*RHO* E3–4, which expresses both His-tagged PRPF31 protein and an RNA splicing template of *RHO* E3–4. Corresponding constructs expressing untagged PRPF31 were generated by first cloning the *PRPF31* cDNA sequence into the vector pTriEx-1 (Novagen) with a stop codon inserted before the His-tag sequence and then transferring the sequence as a *Sac*II-*Xho*I fragment into pTan. *RHO* E3-E4 as previously described.

Full-length *RHO* constructs were generated in a stepwise process because of the relatively large size of the full length *RHO* gene of 4.985 kb (from start to stop codons and including introns). The *RHO* sequence was amplified as two separate fragments (E1-E2 with 5′-*Nhe*I and 3′-*Nae*I tags and E2 –E5 with 5′-*Nae*I and 3′-*Cla*I tags) from human genomic DNA using the following primer pairs: E1F (5′-GCG CGC TAG CAT GAA TGG CAC AGA AGG CCC T-3′) and E2R (5′-GGA CCA GCC GGC GAG TGG-3′); and E2F (5′-CCA CTC GCC GGC TGG TCC-3′) and E5R (5′-CGG CAT CGA TTT AGG CCG GGG CCA CCT G-3′). These two fragments were cloned into the PCR vector pGEM-T Easy (Promega, Southampton, UK). Restriction cloning was then used to ligate the fragments together and transfer the assembled gene sequence into NheI/ClaI-cut pTandem-1. The PRPF31 sequence was inserted as before.

Human *GNAT1* constructs were generated by first transferring the *PRPF31* cDNA sequence into pTandem-1 as described in the previous paragraph and then inserting an exon 2 to exon 6 *GNAT1* fragment amplified from human genomic DNA using *Hpa*I- and *Cla*I-tagged forward and reverse primers respectively (E2F: 5′-GTT AAC ATG GCC GGT GAG TCC-3′ and E6R: 5′-ATC GAT TCA CAC TAG CAC CAT-3′).

**Figure 1:**
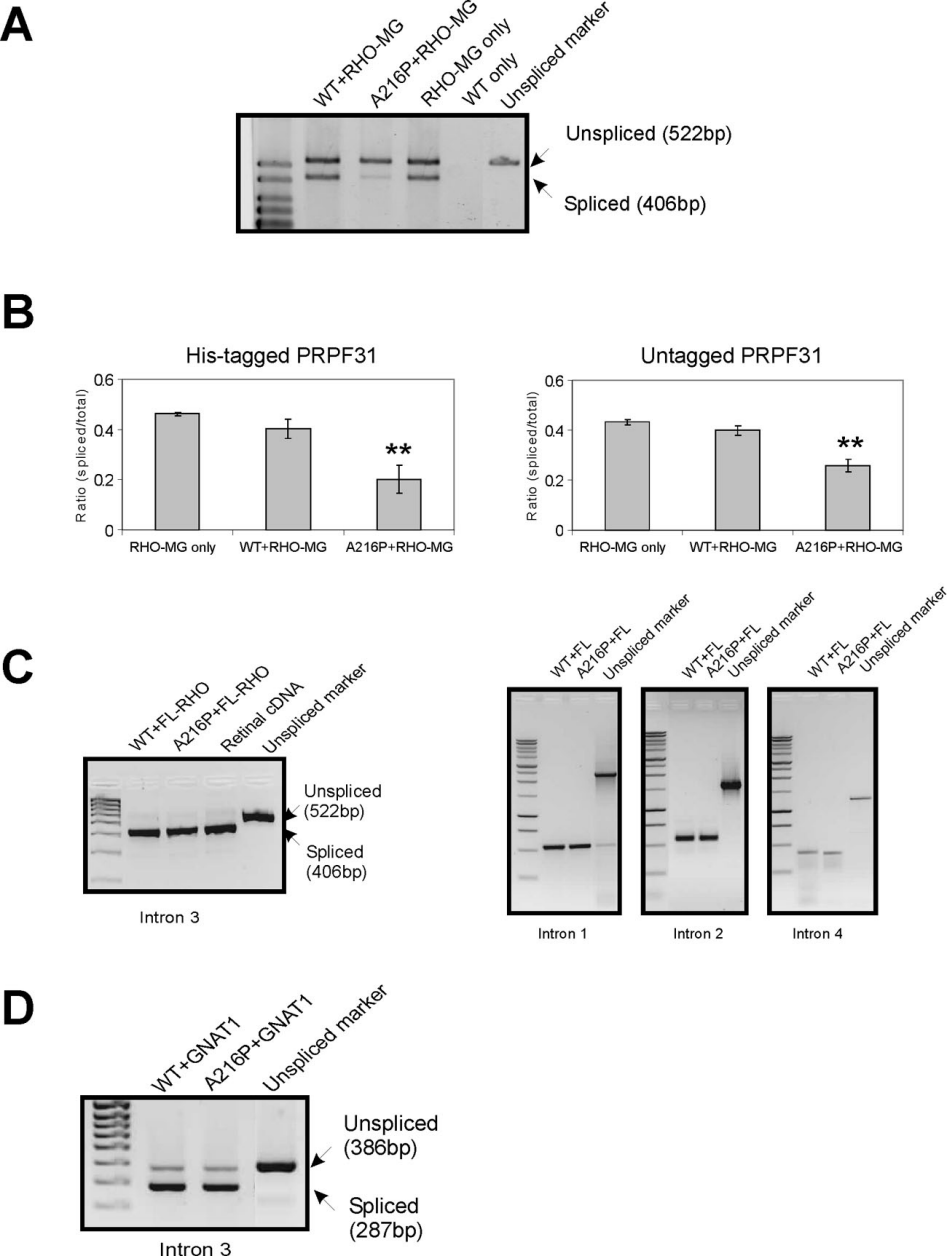
Splicing assays of wild-type and mutant PRPF31 in transfected HEK 293T cells. A: Assays using *RHO* intron 3 minigene splicing template (*RHO*-MG). As a positive control, cells were transfected with the splicing template only (*RHO*-MG only). Cells transfected with WT *PRPF31* only gave no products (negative control). Marker for the unspliced product was generated by amplification directly from the plasmid construct using the same primers. B: Bar graphs show the splicing efficiencies, derived from the relative band strengths for cells expressing untagged and His-tagged PRPF31. Error bars indicate the standard error of means derived from four separate determinations. Double asterisks (**) indicate that the reduced splicing efficiencies of mutant splicing factor compared to the positive and negative controls are statistically significant (p<0.01). C: Assays using full length *RHO* (FL-*RHO*) splicing template. Analysis of all four introns indicates 100% splicing efficiency. No unspliced transcript was detected in cDNA from human retina. D: Assays using *GNAT1* template.

### Plasmid constructs for protein expression

The N-terminal domain (PRPF6N, residues 1–307) and two HAT-containing (half a tetratricopeptide repeat, TPR) domains (PRPF6-M, residues 307–607, and PRPF6-C, residues 607–941) of human *PRPF6* were amplified from human cDNA and inserted into the bacterial expression vector pGEX6P-1 (Amersham Biosciences, Little Chalfont, UK) via the *Eco*RI and *Sal*I sites. The primer pairs used for the amplifications were as follows: for PRPF6N, 5′-GCG CGA ATT CAT GAA CAA GAA CAA ACC GTT CC-3′ and 5′-CAG CGT CGA CTG TCA ATG AGG GTT CGT CTC CCG-3′; for PRPF6M, 5′-GCG CGA ATT CCC CCG CCA GCC TGG ATT GCA TC-3′ and 5′-CAG CGT CGA CTC ATG CTT TGG GGC AGT CGG C-3′; and for PRPF6C, 5′-GCG CGA ATT CCC CGC CAG CCT GGA TTG CAT C-3′ and 5′-GAC GGT CGA CTC AGA AGG TGT TCT TGA TGC G-3′.

### In vivo splicing assays in HEK 293T cells

Human HEK 293T cells in 3.5 cm dishes were transiently transfected using GeneJuice (Novagen) with pTandem-1 constructs which expressed PRPF31 (WT or mutant) and a splicing template comprising either the *RHO* intron 3 mini-gene, the *RHO* full length gene, or the *GNAT1* partial gene. After 48 h, cells were washed twice with PBS and used to prepare total cell RNA using TRIzol reagent (Invitrogen, Paisley, UK). Contaminating DNA carried over from the cell transfection was removed from the extracted RNA by treatment with RNase-free DNaseI (Roche Diagnostics Ltd., Paisley, UK). The RNA was quantified using Quant-iT RiboGreen RNA assay (Invitrogen). RT–PCR was then conducted as follows to assay the relative quantities of spliced and unspliced transcripts from the splicing template. First strand cDNA synthesis was performed with 0.5 µg of total RNA using avian myeloblastosis virus (AMV) reverse transcriptase and oligo-p(dT)15 primer (Roche Diagnostics Ltd). This cDNA was diluted to a total volume of 50 µl, and 5 µl aliquots were used as template in PCR reactions with exonic primer pairs to amplify across the intervening introns. Thermocycling was conducted for the minimum number of cycles (generally 20–25 cycles) to ensure amplification of target sequence remained in the exponential phase. PCR products were analyzed on SYBR Green- (Invitrogen Ltd) or ethidium bromide-stained agarose gels. Relative band strengths were quantified using Genesnap software (Syngene, Cambridge, UK) from gel images.

### Pull-down assays

WT and A216P His-tagged PRPF31 were expressed from pTriEx constructs [15] in *E. coli* Tuner (DE3) pLacI cells (Merck Biosciences Ltd., Nottingham, UK), purified to homogeneity from inclusion bodies under denaturing conditions and subsequently refolded (as described in [16]). Protein concentrations were estimated using a Biorad protein assay kit (Bio-Rad UK Ltd., Hemel Hempstead, UK) against BSA standards.

Glutathione S transferase (GST)-tagged PRPF6 domains (PRP6F-N, -M, -C) were expressed from pGEX6P-1 constructs in *E. coli* BL21 (DE3) cells (Amersham Biosciences). Liquid cultures (Luria-Bertani broth with 100 μg/ml ampicilin) were grown at 37 °C to log phase, induced with 0.1 mM Isopropyl-Beta-D-Thiogalactopyranoside and then grown overnight at 25 °C. Cells were harvested as cell pellets from 20 ml aliquots and soluble extracts containing 1 ml GST-tagged PRP6F-N, PRP6F-M, or PRP6F–C were prepared in PBS buffer by centrifugation of crude cell lysates.

A 100 µl aliquot of GST-tagged PRPF6-N, PRP6F-M, or PRP6F–C extract was first bound to 20 µl of 50% glutathione sepharose beads (Amersham Biosciences) in PBS. Excess PRPF6 was removed, and the beads were washed once with PBS and 0.1 nmol of purified His-tagged PRPF31 was added to give a total volume of 200 μl. The mixture was agitated for 30 min at 4 °C. The beads were then centrifuged and washed three times with chilled PBS. Bound proteins were eluted with 2 × 10 µl aliquots of reduced glutathione buffer. These were analyzed on duplicate western blots, one probed with Penta-His antibodies (Qiagen Ltd, Crawley, UK) and the second with anti-GST antibodies (Merck Biosciences) followed by an HRP-conjugated anti-mouse secondary antibody (Perbio Science UK Ltd., Cramlington, UK). Blots were developed with ECL-Plus HRP detection system (Amersham Biosciences). Relative band strengths were quantified using Genesnap software as described in the previous section. Band quantities were normalized to the strength of the WT band for each determination. Mean values were estimated from four independent determinations.

## Results

### Functional assays of splicing activity

Splicing assays were designed to examine the efficiency of splicing in transfected HEK 293T cells expressing either WT or mutant PRPF31 protein exogenously. These cells also express WT PRPF31 (as well as all the other components of the spliceosome) endogenously. The rationale of the experiment is that the high level of expression of the exogenous splicing factor will compete with the endogenous splicing factor for incorporation into the spliceosome and thus reveal any deleterious effects of the mutations. However, this means that the assays can detect only dominant effects of the mutations and not effects due to insufficiency.

Assays were conducted by transiently expressing PRPF31 protein (WT or mutant) plus a splicing template in HEK 293T cells, extracting total RNA from the cells, and performing RT–PCR to determine the relative quantities of spliced and unspliced RNA products derived from the exogenous splicing template. Previous assays of PRPF31 splicing activity have relied on cotransfection of the cells with two plasmids: one expressing the splicing template and the other PRPF31 [15,17]. This has the drawback that an undefined proportion of the transfected cells will have taken up the splicing template plasmid alone, giving rise to a background of RNA products that have been spliced using only endogenous WT PRPF31. To ensure that all cells expressing the splicing template were also expressing the exogenous PRPF31, plasmid constructs were generated in the pTandem-1 mammalian dual expression vector, which is designed for the coexpression of two open reading frames from a single bicistronic RNA.

### Splicing assays using a human rod opsin RHO intron 3 minigene

A minigene similar to that described by Yuan et al. [17] was constructed that contained a 522 bp sequence amplified across *RHO* exon 3 to exon 4 of human genomic DNA. When pTandem constructs containing this minigene were used to transfect HEK 293T cells, both spliced (406 bp) and unspliced (522 bp) products were generated, indicating inefficient splicing of intron 3 (Figure 1A, lane 4). The splicing efficiency was estimated as the ratio of band fluorescence (after staining with ethidium bromide) for spliced versus total (spliced + unspliced) transcript, with a correction for the relative fluorescence of different size fragments. Splicing efficiency measured in control cells expressing the minigene only, where only the endogenous splicing factor is present, was not affected by addition of exogenous WT PRPF31 (Figure 1A, lane 2), but splicing efficiency was reduced by the presence of A216P mutant PRPF31 (Figure 1A, lane 3). The means±standard error of measurements obtained from four replicate transfections for each experiment with either untagged and His-tagged PRPF31 are shown in Figure 1B. For untagged and tagged PRPF31, therefore, the overall levels of splicing efficiency are similar, indicating that the presence of a His-tag on the exogenous PRPF31 does not affect its splicing function. In both cases, mutant PRPF31 resulted in a statistically significant (p<0.01) reduction in splicing compared to the positive and negative controls..

**Figure 2:**
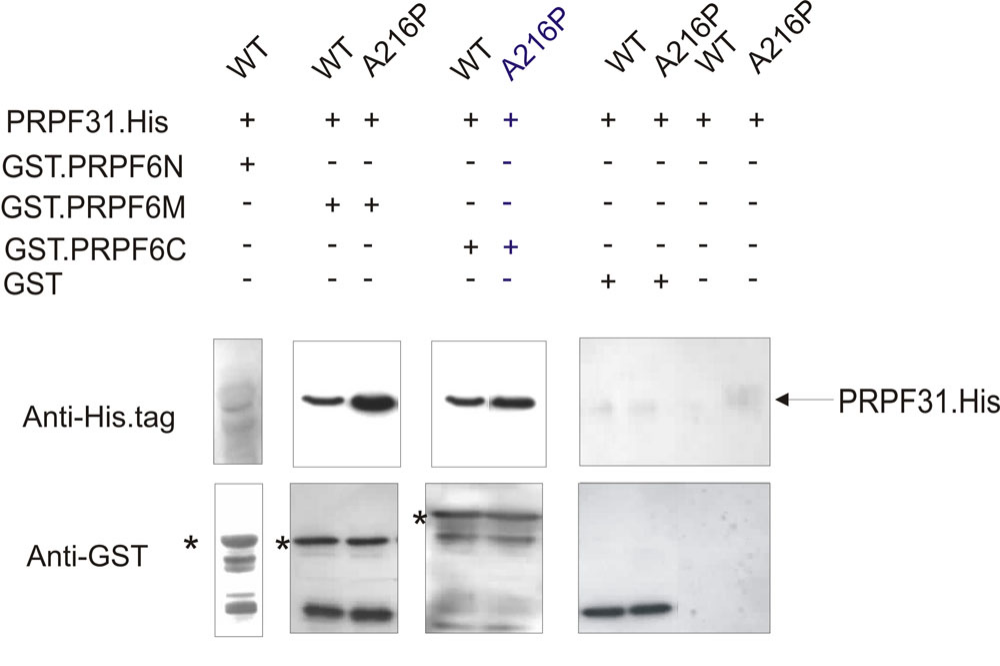
Pull-down assays showing the interaction of His-tagged PRPF31 (WT and A216P mutant) with GST-tagged PRPF6 domains. Complexes between the two proteins were immobilized with glutathione sepharose beads and eluted with reduced glutathione. Panels show the Western analysis of elution products using immobilized GST-PRPF6N (amino acids 1–306), GST-PRPF6M (amino acids 307–607), and GST-PRPF6C (amino acids 607–941). Upper panels were probed with α-His.tag antibody and lower panels with α-GST antibody. Asterisks (*) indicate the size of full-length GST-PRPF6N, GST-PRPF6M, and GST-PRPF6C respectively. Note that the faint band in the upper panel for the pull-down assay with GST-PRPF6N is too small for PRPF31-His. Negative controls show absence of nonspecific binding of His-tagged PRPF31 to the beads and to GST tag. Quantification of the WT and mutant PRPF31 bands gave ratios of mutant:WT of 4.2±1.4 (for PRPF6M) and 2.1±0.5 (for PRPF6C). In each case, the mean and standard error was determined from four separate determinations.

### Splicing assays using full-length human rod opsin template

To ascertain how closely the splicing of a minigene approximates to the splicing of a real pre-mRNA template, we generated transfection constructs that expressed a full-length *RHO* gene from the start to stop codons and included all four introns. RT–PCR was then performed on RNA from transfected cells using primer pairs designed to amplify each intron separately. In contrast to the result with the minigene, the efficiency of splicing of intron 3 with WT exogenous splicing factor was substantially greater, approaching a ratio of 1 for spliced template compared to total template (Figure 1C). When the A216P mutant PRPF31 was used, there was no reduction in splicing efficiency. Results for introns 1, 2, and 4 similarly showed that the cells were able to splice these introns completely even in the presence of mutant PRPF31.

### Splicing efficiency of human rod opsin intron 3 in human retinal tissue

To assess whether the splicing of *RHO* intron 3 was a limiting factor in the production of fully processed *RHO* mRNA in photoreceptors, we generated PCR-amplified fragments using an exon 3 forward and exon 4 reverse primer pair from the *RHO* transcript in mRNA obtained from human retinal tissue. As shown in Figure 1C, no unspliced product was obtained, similar to our experiments with the full-length *RHO* template, indicating that the latter splicing assays may model the situation in rod photoreceptor cells more closely than assays with minigenes.

### Splicing assays using partial GNAT1 template

It has been suggested that small introns may be spliced less efficiently than larger introns [17]. The human *GNAT1* gene has several small introns of lengths between 89 and 210 bp. We therefore generated splicing constructs containing a partial gene of *GNAT1* that comprised exons 2 to 6 and intervening introns, representing approximately one-third of the genomic sequence. Splicing assays using these constructs indicated that introns 3 and 5 are inefficiently spliced. However, no difference in splicing efficiency was detected when WT or A216P mutant PRPF31 was co-expressed (results for intron 3 shown in Figure 1D).

**Figure 3:**
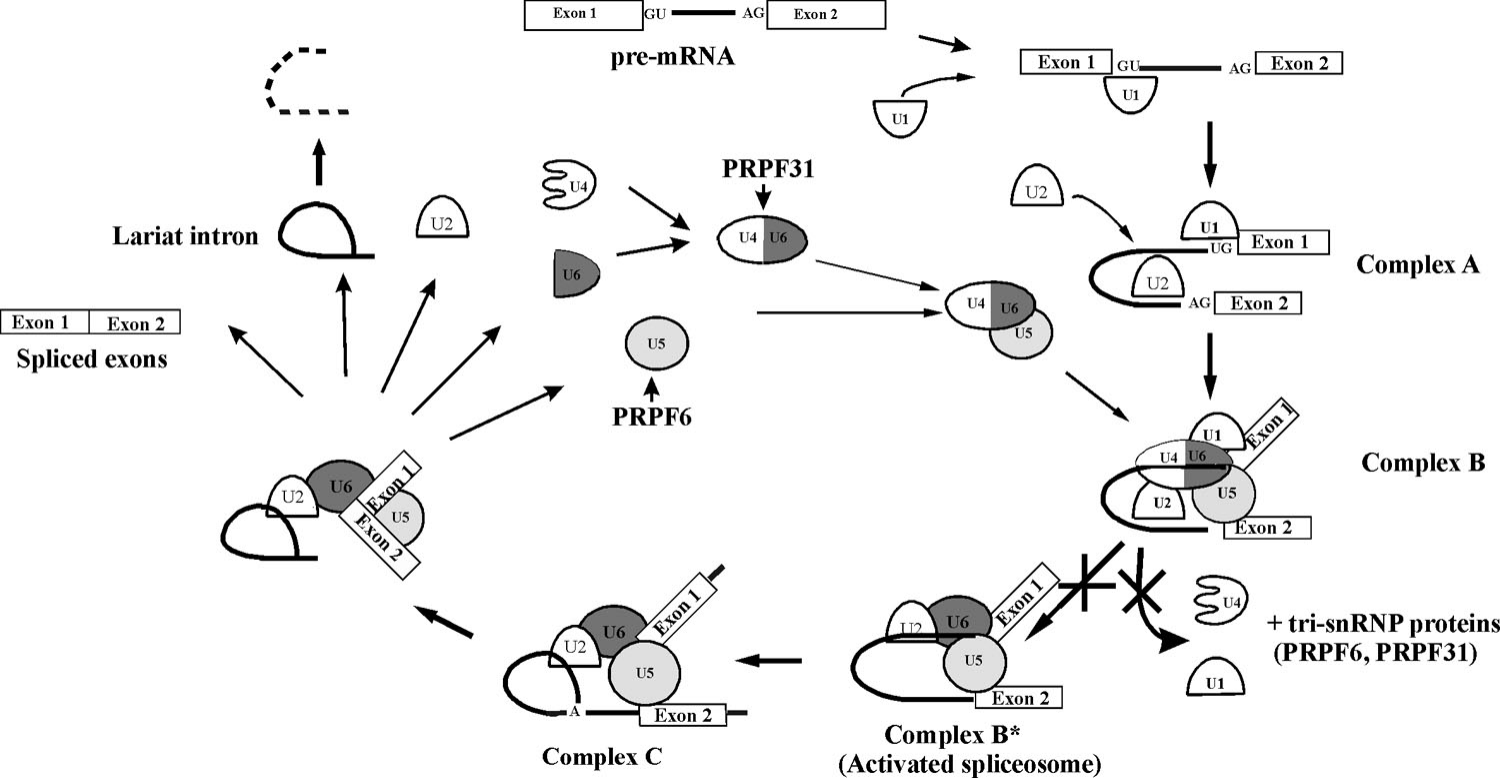
Role of PRPF31 in pre-mRNA splicing. PRPF31 is a splicing factor specific to the U4/U6 snRNP, and its primary role is to recruit and physically tether U5 to U4/U6 to yield the U4/U6+U5 tri-snRNP. This involves an interaction between PRPF31 and the U5-specific splicing factor PRPF6. Once incorporated into the spliceosomal complex B, activation involves loss of U1 and U4 and all tri-snRNP associated proteins, including PRPF31 and PRPF6. The mutant PRPF31 proteins are predicted to inhibit this activation step (marked with crosses) and prevent recycling of spliceosomal components for future splicing events.

### Interaction of wild-type and mutant PRPF31 with PRPF6

Formation of the U4/U6+U5 tri-snRNP component of the spliceosome involves an interaction between PRPF31 (a component of U4/U6) and PRPF6 (a component of U5) as demonstrated by biochemical pull-down experiments and yeast two-hybrid studies [21,23]. The PRPF6 protein contains an N-terminal domain followed by several repeat motifs of loose consensus classified as HAT domains, a feature present in many RNA binding proteins [24]. Pfam analysis [25] identified 9 such motifs. The interaction of PRPF6 with PRPF31 was initially reported to involve the C-terminal HAT repeats only [21] but has more recently been shown to involve other HAT repeats [23].

In this study, we expressed PRPF6 protein as three separate domains comprising residues 1–307 (PRPF6N), 307–607 (PRPF6M, HAT domains 1–5 according to Pfam analysis) and 607–941 (PRPF6C, HAT domains 6–9 according to Pfam analysis), together with N-terminal GST tags. Pull-down experiments with His-tagged WT PRPF31 indicated interactions with PRPF6M as well as PRPF6C, but not with PRPF6N (Figure 2A), confirming previous results [21,23]. Quantitative experiments using equivalent quantities of purified WT and mutant PRPF31 proteins indicated that the interaction of both PRPF6M and PRPF6C was substantially stronger with mutant than with WT PRPF31 (Figure 2B).

## Discussion

The results presented in this study demonstrate that the A216P missense mutation in *PRPF31* has a dominant deleterious effect on the splicing of *RHO* intron 3 transcribed from a minigene in transfected mammalian cells. Using a similar minigene assay, Yuan et al. [17] reported a comparable deleterious effect in cells expressing truncated PRPF31 proteins based on two pathogenic frameshift mutations (RP11 families SP117 and AD5 [4]). However, unlike the missense mutations, where mutant proteins will almost certainly be expressed in the cells of affected patients, such truncated proteins may either be rapidly degraded or not produced due to nonsense mediated decay (NMD). This latter process results in the rapid removal of mutant transcripts that contain a premature termination codon (reviewed in [26]).

The results of the pull-down assays described here provide a possible explanation for the dominant deleterious effect of the missense mutation on function. Our results show that the A216P mutation results in a stronger interaction between the PRPF31 and HAT-motifs in PRPF6. This will stabilize the U4/U6+U5 snRNP and should promote the formation of splicing complex B but could inhibit subsequent protein-RNA movements during the later stages of the splicing cycle. In particular, activation of the spliceosomal complex B is believed to involve extensive remodeling that includes the removal of U4 and all tri-snRNP proteins, including PRPF6 and PRPF31 (see Figure 3, modified from [27]). In effect, once incorporated into a spliceosomal complex, the mutant protein may inhibit its activation for that splicing event and the subsequent recycling of the spliceosomal components for future splicing events.

While the observation of a dominant deleterious effect of the A216P missense mutation in PRPF31 could account for the dominant nature of the RP11 form of disease, it fails to offer an explanation for the incomplete penetrance of the disorder in families with obligate carriers of the disease allele. Reduced splicing efficiency could only be detected with minigene transcripts, not full-length transcripts from a complete gene sequence; splicing from a full-length transcript appears to occur therefore with full efficiency in the presence of mutant splicing factor. One possible explanation for this discrepancy is that greater splicing efficiency may be achieved with a full-length transcript, because some intrinsic feature in the RNA facilitates splicing or promotes binding of an extrinsic splicing enhancer. For example, splicing enhancers may show exonic binding (reviewed in [28]) and will not function in a minigene assay. Whatever the mechanism, the assays using the full-length gene construct are more likely to reflect conditions pertaining in photoreceptor cells than those using minigenes. This is not to say that the observed splicing deficiency using the minigene assays is an artifact, rather we suggest that the design of the assay is such as to reveal a rather mild effect of the mutation. Severe RP arises from haploinsufficiency but can be prevented if a high-expressing WT allele is present. Such unaffected carriers of RP11 mutations [29] may nevertheless show a mild retinal pathology that may be attributable to a residual haploinsufficiency. However, it may also arise from a reduction in the efficiency of splicing caused by the mutant PRPF31 protein.

Our results using the *GNAT1* splicing template support previous suggestions that splicing of small introns may be less efficient than splicing of larger ones [17]. Splicing of both introns 3 and 5 was inefficient, even with a transcript containing multiple exons and introns. However, even with these weak introns, our assays failed to detect any effect of the A216P mutation on splicing efficiency. This is consistent therefore with our result with the full-length *RHO* transcript.

Our previous work has shown that another effect of the missense mutations is to reduce protein solubility and cause protein mislocation when expressed in transfected cells [15,16]. This will result in substantially reduced levels of functional PRPF31 in the nucleus, which may in turn lead to an accumulation of unspliced transcripts and aberrant proteins, eventually causing cell death. The pathological mechanism whereby *PRPF31* missense mutations result in RP is therefore protein insufficiency, and this may also underlie the disease pathology of other *PRPF31* mutations studied to date, including gene deletion [7,10], where no mutant protein can be present, and mutations leading to premature termination where NMD of mutant transcripts occurs [13]. Based on the results presented in this report, we have now found that the missense mutations have a direct effect on splicing efficiency in addition to the proposed more general effect on PRPF31 sufficiency, thereby creating a second mechanism for the pathology. However, it is probable that the specific effect on splicing makes only a minor contribution to the development of the disease, since it could not be detected using either a full-length *RHO* template or a partial *GNAT1* template. The major effect of these mutations remains therefore a reduction in the amount of PRPF31 protein entering the nucleus.
